# Microalgae and Cyanobacteria Strains as Producers of Lipids with Antibacterial and Antibiofilm Activity

**DOI:** 10.3390/md19120675

**Published:** 2021-11-27

**Authors:** Virginio Cepas, Ignacio Gutiérrez-Del-Río, Yuly López, Saúl Redondo-Blanco, Yaiza Gabasa, María José Iglesias, Raquel Soengas, Andrés Fernández-Lorenzo, Sara López-Ibáñez, Claudio J. Villar, Clara B. Martins, Joana D. Ferreira, Mariana F. G. Assunção, Lília M. A. Santos, João Morais, Raquel Castelo-Branco, Mariana A. Reis, Vitor Vasconcelos, Fernando López-Ortiz, Felipe Lombó, Sara M. Soto

**Affiliations:** 1 ISGlobal, Hospital Clínic—Universitat de Barcelona, 08036 Barcelona, Spain; virgicepas5@gmail.com (V.C.); yulydelosangeles@gmail.com (Y.L.); yaiza.gabasa@isglobal.org (Y.G.); 2 Research Group BIONUC (Biotechnology of Nutraceuticals and Bioactive Compounds), Departamento de Biología Funcional, Área de Microbiología, Universidad de Oviedo, 33006 Oviedo, Spain; nachogutiem@gmail.com (I.G.-D.-R.); redondo.saul@gmail.com (S.R.-B.); andresfernandezlorenzo92@gmail.com (A.F.-L.); saralopezibanez@gmail.com (S.L.-I.); cjvg@uniovi.es (C.J.V.); lombofelipe@uniovi.es (F.L.); 3 IUOPA (Instituto Universitario de Oncología del Principado de Asturias), Principality of Asturias, 33006 Oviedo, Spain; 4 ISPA (Instituto de Investigaciones Sanitarias del Principado de Asturias), Principality of Asturias, 33011 Oviedo, Spain; 5 Área de Química Orgánica, Centro de Investigación CIAIMBITAL, Universidad de Almería, 04120 Almería, Spain; mjigle@ual.es (M.J.I.); rsoengas@ual.es (R.S.); flortiz@ual.es (F.L.-O.); 6 Coimbra Collection of Algae (ACOI), Department of Life Sciences, Calçada Martim de Freitas, University of Coimbra, 3000-456 Coimbra, Portugal; martinscsb@gmail.com (C.B.M.); ferreirajoanadias@gmail.com (J.D.F.); mariana.assuncao@uc.pt (M.F.G.A.); liliamas@ci.uc.pt (L.M.A.S.); 7 “Molecular Physical-Chemistry” R&D Unit, Department of Chemistry, University of Coimbra, 3004-535 Coimbra, Portugal; 8 Interdisciplinary Centre of Marine and Environmental Research (CIIMAR/CIMAR), Terminal de Cruzeiros do Porto de Leixões, University of Porto, 4450-208 Matosinhos, Portugal; joaopmorais@gmail.com (J.M.); raquelcastelobranco12@gmail.com (R.C.-B.); mreis@ciimar.up.pt (M.A.R.); vmvascon@fc.up.pt (V.V.); 9 Faculdade de Ciências, Universidade do Porto, Rua do Campo Alegre, Edifício FC4, 4169-007 Porto, Portugal

**Keywords:** microalgae, cyanobacteria, free fatty acids, glycoglycerolipids, phospholipids, antimicrobial, antibiofilm, human pathogens

## Abstract

Lipids are one of the primary metabolites of microalgae and cyanobacteria, which enrich their utility in the pharmaceutical, feed, cosmetic, and chemistry sectors. This work describes the isolation, structural elucidation, and the antibiotic and antibiofilm activities of diverse lipids produced by different microalgae and cyanobacteria strains from two European collections (ACOI and LEGE-CC). Three microalgae strains and one cyanobacteria strain were selected for their antibacterial and/or antibiofilm activity after the screening of about 600 strains carried out under the NoMorFilm European project. The total organic extracts were firstly fractionated using solid phase extraction methods, and the minimum inhibitory concentration and minimal biofilm inhibitory concentration against an array of human pathogens were determined. The isolation was carried out by bioassay-guided HPLC-DAD purification, and the structure of the isolated molecules responsible for the observed activities was determined by HPLC-HRESIMS and NMR methods. Sulfoquinovosyldiacylglycerol, monogalactosylmonoacylglycerol, sulfoquinovosylmonoacylglycerol, α-linolenic acid, hexadeca-4,7,10,13-tetraenoic acid (HDTA), palmitoleic acid, and lysophosphatidylcholine were found among the different active sub-fractions selected. In conclusion, cyanobacteria and microalgae produce a great variety of lipids with antibiotic and antibiofilm activity against the most important pathogens causing severe infections in humans. The use of these lipids in clinical treatments alone or in combination with antibiotics may provide an alternative to the current treatments.

## 1. Introduction

Nowadays, antimicrobial resistance (AMR) is one of the major public health challenges. The alarming decline in the effectiveness of antibiotic treatments due to the increasing resistance acquired by pathogens has put the world in a global AMR crisis [1,2]. In addition, biofilm formation makes these bacteria up to 1,000-fold more resistant to antibiotics. Therefore, there is an urgent need to find new compounds to solve this problem, and one of the neglected ecological niches to look for new bioactive compounds against pathogen bacteria is the ocean, which covers approximately 71% of Earth’s surface. Indeed, aquatic environments are some of the most promising places for the discovery of new antibiotics and antibiofilm compounds due to their extraordinary biodiversity, from microscopic to macroscopic organisms. 

Microalgae (photosynthetic eukaryotes) and cyanobacteria (photosynthetic prokaryotes) represent a large part of the biodiversity of these aquatic environments as they are at the base of their food chain [3,4]. These microorganisms can adapt to many environments, including extreme ones characterized by the presence of toxic substances or extreme temperature, values of pH, or salinity. This feature enables the development of different defense mechanisms and production of bioactive compounds that allow them to survive in such extreme conditions [2,4,5,6]. The chemical diversity of these metabolites, combined with their activity inhibiting multiple tolerant pathogens’ growth [3], place these microorganisms at the forefront of drug discovery (the process associated with the screening and discovery of bioactive molecules and their subsequent development as pharmaceuticals) and antimicrobial resistance research.

Therefore, although drug development can be challenging, new drugs can be developed from secondary metabolites of microalgae and cyanobacteria, such as dolastatin, the only currently approved drug derived from cyanobacteria (*Symploca hydnoides* and *Moorea producens*, formerly *Lyngbya* *majuscula*) [7,8,9]. The antibiotic activity of microalgae and cyanobacteria has been attributed to compounds belonging to several chemical classes, such as alkaloids, aromatic compounds, acetogenins, cyclophanes and paracyclophanes, dicarboximides, indanes, indoles, lactones, lipids, macrolides, phenols, phlorotannins, peptides, pigments, polyphenyl ethers, polysaccharides, porphinoids, sterols, and terpenes [1,2,3,4,5,6,7,9]. Considering all this chemical diversity, this work will focus on antimicrobial lipids, which can generally be defined as lipid amphiphiles that interact with and destabilize bacterial cell membranes, and they are attractive candidates for exploration as new broad spectrum antibacterial agents to fight against bacterial infections [10,11,12]. They can be classified into three broad categories: fatty acids (hydrocarbon chains with a carboxylic acid functional group), glycoglycerolipids (composed of glycerol, fatty acids, and carbohydrates), and monoglycerides (esterified adducts of a fatty acid and glycerol molecule) [11]. 

Fatty acids are ubiquitous in nature and can be defined as organic compounds with carboxylic acids and long aliphatic chains that can be straight or branched, as well as saturated, unsaturated, or hydroxylated. Their multiple biological properties, including antibiotic properties, are well-known [13,14,15]. In fact, the antibacterial effects of fatty acids were first described in the 1880s by Dr. Robert Koch and colleagues who observed that fatty acids inhibited the growth of *Bacillus anthracis*. However, these compounds lost importance due to the golden age of antibiotics, but they have now regained prominence with the growing impact of antibiotic-resistant bacteria [10]. 

The potent antibacterial effect described for fatty acids led to studies focused on the use of omega-3 polyunsaturated fatty acids, such as docosahexaenoic acid (DHA) and eicosapentaenoic acid (EPA), as antibacterial and antibiofilm agents against periprosthetic joint infections caused by multi-drug resistant strains (*Staphylococcus epidermidis*, *S. aureus,* and coagulase-negative *Staphylococci*) [16,17]. In addition, EPA and DHA have been reported as having the ability to bind to non-specific proteins, and this non-specific mechanism of action is very beneficial in reducing antibiotic resistance [17]. These studies provide increasing evidence for the use of fatty acids as an alternative to classical antibiotics, or in combination with them, to enhance their effects [18].

The multiple mechanisms that confer fatty acids their antibacterial activity are not yet fully understood, although it is known that these compounds are released to the environment when the cell loses its integrity, and they act as a defense mechanism to protect bacterial populations from grazing predators or pathogenic bacteria [19]. It seems that bacterial cell membranes could be the principal target of polyunsaturated fatty acids (such as DHA or EPA) or saturated fatty acids (such as lauric acid) through the insertion of their long carbon chains, thereby causing cell leakage, reducing nutrient intake, and affecting cellular respiration [12,19,20,21,22,23]. 

As a general rule, cyanobacteria and microalgae contain high amounts of proteins, with values even higher than soybean, corn, and wheat. Of particular interest is also their content of polyunsaturated fatty acids, including α-linolenic acid (ALA), γ-linolenic acid (GLA), EPA, DHA, and arachidonic acid (ARA). Although lipid accumulation is extremely dependent on the culture conditions, the lipid content of some microalgae species, such as *Nannochloropsis oceanica*, *Chlorella vulgaris,* or *Scenedesmus obliquus,* can represent more than 20% of their dry weight [24]. The first compound with antibiotic activity isolated from microalgae was described by Pratt et al. in 1944, and it was purified from chloroform and benzene extracts of *Chlorella vulgaris* as a mixture of fatty acids. It was named chlorellin and was able to inhibit the growth of both Gram-positive (*Bacillus subtilis*, *Staphylococcus aureus,* and *S. epidermidis*) and Gram-negative (*Escherichia coli* and *Pseudomonas aeruginosa*) bacteria [25,26]. Since then, the interest in microalgae as potential antibiotic producers has led to several large screening programs to investigate the potential of different freshwater and marine taxonomic groups of microalgae to inhibit the growth of pathogenic and foodborne bacteria [27,28,29,30,31]. Some of the antibacterial compounds present in the extracts were characterized and, in most cases, these were free fatty acids. Short chain fatty acids from *Haematococcus pluvialis* [32] and long chain fatty acids from *Scenedesmus obliquus* [33] have been shown to exhibit antibacterial activity against *S. aureus* and *E. coli*. On the other hand, it was found that polyunsaturated fatty acids from *Chlorococcum* strains HS-101 and *Dunaliella primolecta* were active against methicillin-resistant *S. aureus* (MRSA) [34]. It has also been described that polyunsaturated fatty acid (EPA), monounsaturated fatty acid (palmitoleic acid, PA), and polyunsaturated fatty acid (hexadecatrienoic acid, HTA) from the diatom *Phaeodactylum tricornutum* are active against MRSA [35,36]. Finally, the antibiotic activity of supercritical CO_2_ extracts from the microalga *Chaetoceros muelleri* has been related to the lipid composition [37]. 

Glycoglycerolipids (GLs) represent a neglected class of metabolites of increasing interest. Nevertheless, their low natural abundance coupled with their difficult isolation makes it hard to evaluate their bioactivities, which include antibacterial, antiviral, anti-tumor, and anti-inflammatory activities [38]. These compounds are especially abundant in microalgae, macroalgae (macroscopic algae), and cyanobacteria and exhibit a glycerol backbone that anchors one or two acyl chains esterified at the *sn*-1 and *sn*-2 positions, and a sugar group attached at the *sn*-3 position in a β-anomeric linkage [39]. GLs can be classified into three broad classes according to the nature of the glycosidic head: monogalatosyldiacylglycerols (MGDGs), digalactosyldiacylglycerols (DGDGs) (and their monoacylated forms, monogalactosylmonoacylglycerols (MGMGs), and digalactosylmonoacylglycerols (DGMGs)), and sulfoquinovosyldiacylglycerols (SQDGs) (and their monoacylated form sulfoquinovosylmonoacylglycerols, SQMG) [39]. The former two are neutral glycolipids, and the latter are anionic sulfolipids [40]. The biological activities of GLs are known to be dependent on sugar moiety and acyl chains; however, the specific structure-activity relationship is not yet fully understood [38]. 

GLs are mainly located in the plastid and thylakoid membranes of the chloroplasts of eukaryotic algae and perform important functions related to the fluidity and stabilization of photosynthetic apparatus membranes, as well as photoprotection mechanisms involving the xanthophyll cycle. The membrane composition of chloroplasts (and, therefore, cyanobacteria) is highly conserved, with MGDG and DGDG being the most abundant lipids [41,42]. They are not only interesting because of their powerful surfactant and emulsifying properties but also because they are biodegradable and environmentally non-toxic. In fact, because they are odorless, tasteless, and non-irritants, they have interesting applications in the food industry and in cosmetic formulations [43]. 

Finally, another important class of lipids is phospholipids. They are the component of all cell membranes and are synthesized by both prokaryotic and eukaryotic organisms, playing important structural and metabolic roles in these cells. Most are characterized by a common backbone of phosphatidic acid (PA), formed from L-glycerol 3-phosphate with two fatty acids esterified on positions one and two. The algae contain three major phospholipids: phosphatidylglycerol (PG), phosphatidylethanolamine (PE), and phosphatidylcholine (PC), which show diverse biological antitumoral, antiviral, and antibacterial activities [44]. Among these, lysophosphatidylcholine (LPC) is a chemotactic factor that stimulates immune cells and regulates the balance between the release of pro- and anti-inflammatory cytokines. It has been reported that the pretreatment of bacterial infections with LPC showed beneficial effects, preventing the release of proinflammatory cytokines and increasing the release of anti-inflammatory cytokines, and, therefore, helping to eradicate the infection [45].

In this work, the isolation and structural elucidation, as well as the antibiotic and antibiofilm activities, of diverse lipids produced by different microalgae and cyanobacteria strains were studied. These molecules can be used for coating prosthetic devices to avoid bacterial adhesion and posterior biofilm-related infection, or as new antibiotics or adjuvants.

## 2. Results

### 2.1. Microalgae and Cyanobacteria Strains Selection

The microalgae and cyanobacteria strains from the Coimbra Collection of Algae (ACOI) and the Blue Biotechnology and Ecotoxicology Culture Collection (LEGE-CC) collections were selected among 600 strains after the screening of the antibiotic and antibiofilm activities of total cellular extracts in the NoMorFilm H2020 project. The extracts were obtained from freeze-dried biomasses sequentially extracted with hexane, ethyl acetate, and methanol [8]. These strains belonged to the phyla *Cercozoa*, *Charophyta*, *Chlorophyta*, *Cryptophyta*, *Cyanobacteria*, *Euglenophyta*, *Glaucophyta*, *Haptophyta*, *Miozoa*, *Ochrophyta*, *Rhodophyta*, and two unknown species [8]. The antibiofilm and antibacterial activities of these extracts were analyzed against *S. aureus*, *S. epidermidis*, *K. pneumoniae*, *E. cloacae*, *P. aeruginosa*, *E. coli*, *C. parapsilosis*, *C. albicans,* and Coagulase-negative *Sthaphylococcus* (CoNS) strains, and those showing both activities were further studied. 

### 2.2. Antibiotic Activity of Fractions and Sub-fractions

Among the different solid phase extraction (SPE) fractions and high-performance liquid chromatography with diode-array detection (HPLC-DAD) sub-fractions assayed, only eight of them showed an antibiotic effect against the bacterial pathogen *S. aureus* (Table 1). No activity was found against the other eight bacterial species included in the study.

### 2.3. Antibiofilm Activity of Fractions and Sub-Fractions

Among the extracts assayed, those from the methanol (C) and ethyl acetate (B) extraction were the most active against the microorganisms under study. The sub-fraction showing the antibiofilm activity of each strain presented a biofilm inhibition of 60% for CoNS, *E. coli,* and *C. albicans* in the case of ACOI 118_C8_F48-F49; 80% of inhibition for CoNS and *E. coli* in the case of ACOI 1261_C8_F26-F27 and F47-F51, respectively; and 61% and 70% of biofilm inhibition in CoNS in the case of the sub-fractions F34 and F48 of *Sphaerospermopsis* sp. LEGE00249, respectively (Table 2).

### 2.4. HRESIMS and NMR Results

**Figure 1:**
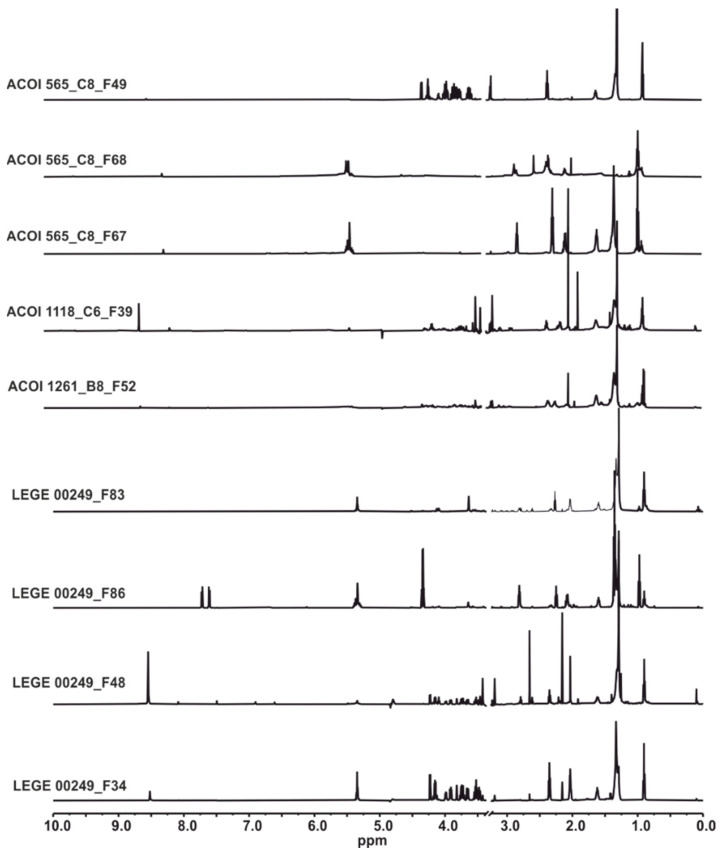
^1^H NMR (600.13 MHz) spectra of the fractions analyzed.

The structure of the compounds responsible for the antibiotic and antibiofilm activities was elucidated based on the combination of spectroscopic and spectrometric methods (Figure 1). 

**Figure 2:**
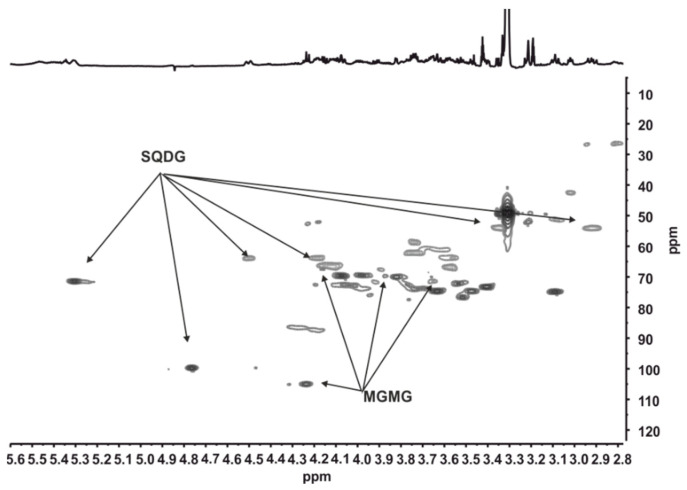
Expansion of the HSQC (600.13 MHz) spectrum of the ACOI 1261_B8_F52 fraction measured in CD_3_OD.

The sulphoquinovosyl backbone in SQDG and SQMG was identified by ^1^H NMR by the anomeric proton (δ_H_ 4.77 ppm, d *J* 3.7 Hz; δ_C_ 99.8 ppm) and the two diasterotopic protons of the methylene group attached to the sulphonyl moiety. These protons resonate at δ_H_ 2.93 ppm (dd *J* 14.4 Hz, 9.1 Hz) and 3.37 ppm and showed a correlation in the HSQC spectrum with the carbon at δ_C_ 53.9 ppm. In the sample ACOI 1261_B8_F52, the *sn*-1 (δ_H_ 4.51, 4.19 ppm/ δ_C_ 63.9 ppm) and *sn*-2 (δ_H_ 5.32 ppm/ δ_C_ 71.4 ppm) signals of the glycerol in SQDG were unambiguously identified [46]. The lack of the characteristic signal of sn1 of glycerol in SQDG in ACOI 118_C6_F39 indicated that the glycerol moiety attached to sulphoquinovose was partially hydrolyzed. The inspection of the HSQC spectrum and comparison with the data from the literature evidenced the presence of SQMG hydrolyzed at the *sn*-2 position of the glycerol [47]. In addition, the ^1^H NMR spectrum of the ACOI 1261_B8_F52 sub-fraction also showed the signal of the anomeric proton of β-galactosyl residue (δ_H_ 4.237 ppm, d *J* 7.7 Hz; δ_C_ 105.3 ppm). Based on the correlations in the HSQC spectrum and the comparison of the ^1^H and ^13^C chemical shifts with those reported in the literature [46], this proton was assigned to MGMGs in which the acyl chain is attached to the sn1 of the glycerol. The nature of the acyl chains attached to glycerol moiety in these compounds could not be unambiguously determined by ^1^H NMR, but it was deduced through the HRESIMS analysis. The mass spectrum of ACOI 1261_B8_F52 displayed peaks at *m*/*z* 821.5456 and 491.3227. The ion at *m*/*z* 821.5456 was consistent with a sulphoquinovosyldiacylglycerol bearing C18:0 and C16:0 acyl chains and C18:0/C16:0 SQDG ([M]^−^, calcd. for C_43_H_81_O_12_S^−^: 821.5454), whereas that at *m*/*z* 491.3227 was identified as 1-palmitoyl-3-O-galactosyl-sn-gycerol, C16:0 MGMG, ([M − H]^−^, calcd. for C_25_H_47_O_9_: 491.3220). On the other hand, the mass peak at *m*/*z* 555.2842 for ACOI 118_C6_F39 allowed 1-palmitoyl-3-O-sulphoquinovosyl-sn-gycerol, C16:0 SQMG, to be identified ([M]^−^, calcd. for C_25_H_47_O_11_S^−^: 555.2845) (Figure 2).

MGMGs were also detected as the major compounds in LEGE 00249_F34 and LEGE 00249_F48. The analysis of the δ 3.0-0.5 ppm region in the ^1^H NMR spectra of these samples indicated that MGMGs differ in the acyl chain, are monounsaturated in LEGE 00249_F34, and saturated in LEGE 00249_F48. In fact, the HRESIMS data of LEGE 00249_F48 confirmed the presence of C16:0 MGMG also found in ACOI 1261_B8_F52 (see above). The peak at *m*/*z* 489.3070 ([M − H]^−^, calcd for C_25_H_45_O_9_: 489.3064) found in the mass spectrum of LEGE 00249_F34 indicated the presence of a palmitoleyl moiety. 

The existence of free fatty acids, such as α-linolenic acid (ALA, C18:3 ω-3), hexadeca-4,7,10,13-tetraenoic acid (HDTA, C16:4 ω-3), and palmitoleic acid (POA, C16:1 ω-7), in some of the sub-fractions studied (Table 1) was established by HRESIMS through the ions at *m*/*z* 277.2176 ([M − H]^−^, calcd. for C_18_H_29_O_2_: 277.2168), *m*/*z* 247.1704 (([M − H]^−^, calcd. for C_16_H_23_O_2_,: 247.1698), and *m*/*z* 253.2176 ([M − H]^−^, calcd. for C_16_H_29_O_2_: 253.2168), respectively. These three free fatty acids were also unambiguously identified by ^13^C NMR spectroscopy [48,49].

**Figure 3:**
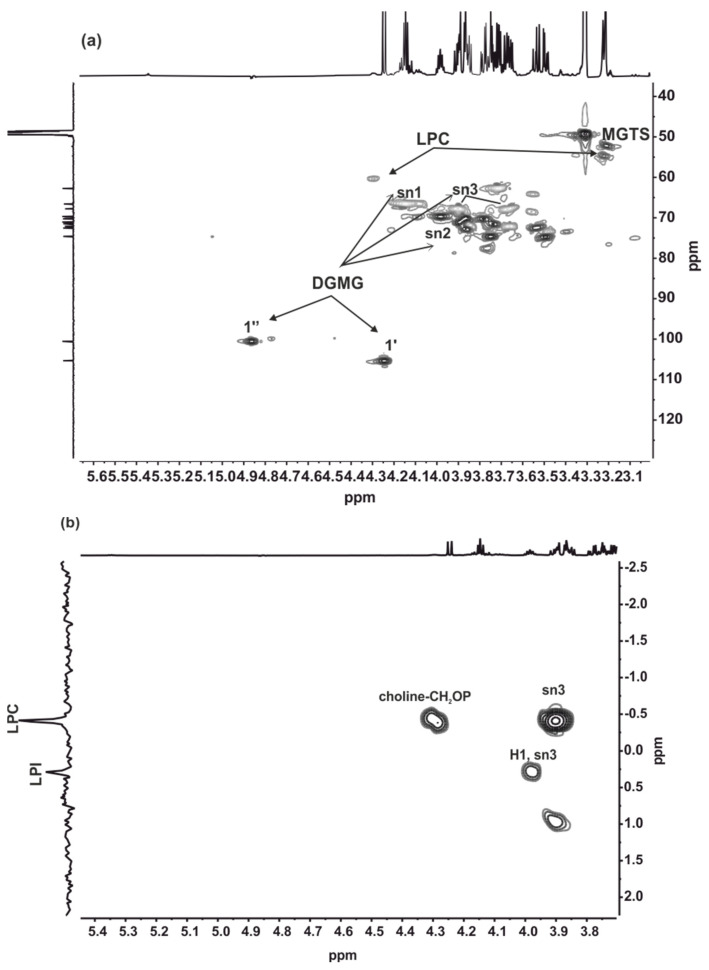
Expansion of the HSQC (600.13 MHz) (**a**) and ^1^H, ^31^P HMQC (600.13 MHz) (**b**) spectra of ACOI565_C8_F49 measured in CD_3_OD.

The analysis of the NMR spectra suggested a similar composition for the ACOI 565_C8_48 and ACOI 565_C8_49 samples. A detailed NMR study of ACOI 565_C8_49 provided the identification of the major compounds. Signals at δ_H_ 4.24 ppm (d *J* 7.0 Hz), δ_C_ 105.3 ppm, and δ_H_ 4.87 ppm (d *J* 3.8 Hz), δ_C_ 100.6 ppm were assigned to the anomeric protons of the sugar moiety in sn-2 digalactosymonoacyl glycerols (DGMG) ) (1’ and 1’’, respectively, in Figure 3a) [50]. Furthermore, the ^13^C spectrum of this sub-fraction determined the esterification with palmitoyl acid (C16:0). In agreement with this, the HRESIMS showed a major peak at *m*/*z* 653.3754 (([M − H]^−^, calcd for C_31_H_57_O_14_: 653.3748). 

The presence of 1-stearoyl-*sn*-glycero-3-phosphocholine (C18:0 LPC) in this sub-fraction was deduced from the HRESIMS spectrum showing a peak at *m*/*z* 522.3562 ([M − H]^−^, calcd. for C_26_H_53_NO_7_P: 522.3560). A similar structure with a shorter aliphatic chain, 1-palmitoyl-sn-glycero-3-phosphocholine (C16:0 LPC), was found to have the molecular formula C_24_H_50_NO_7_P according to the HRESIMS spectrum showing a peak at *m*/*z* 494.3255 ([M − H]^−^ calcd. for C_24_H_49_NO_7_P: 494.3247). The ^31^P{^1^H} NMR spectrum consisted of two signals at δ_P_ 0.29 and -0.42 ppm, which were assigned to lysophosphatidylinositol, 1-oleyl-*sn*-glycero-3-phosphatylinositol (C18:1 LPI) according to the *m*/*z* 597.3046 ([M]^−^, calcd. for C_27_H_50_O_12_P^−^: 597.3045), and lysophsophatidylcholine, respectively [51,52,53]. The lack of correlations in the ^1^H, ^31^P HMQC spectrum (Figure 3b) between the phosphorous signals and protons resonances at ca. δ_H_ 5.25 ppm evidenced that the position *sn*-2 of the glycerol was not esterified. The HRESIMS spectrum also identified C16:0 SQMG in the mixture (Table 2). However, the relative proportion of this compound was below the detection level of ^1^H NMR spectroscopy. The NMR analysis of the ACOI 565_C8_49 subfraction also identified monoacyl-*N,N,N*-trimethylhomoserine (MGTS). However, the nature of the acyl chain could not be determined. The identification of MGTS (Figure 3a) was achieved by the methyl resonance of the tetramethylammonium group at δ_H_ 3.21 ppm/δ_C_ 52.4 ppm, the correlations found in the 2D spectra, and the lack of the resonances for the sn2 position of the glycerol in DGTS at δ_H_ 5.26 ppm/δ_C_ 71.7 ppm [54] (Figure 3). 

**Figure 4:**
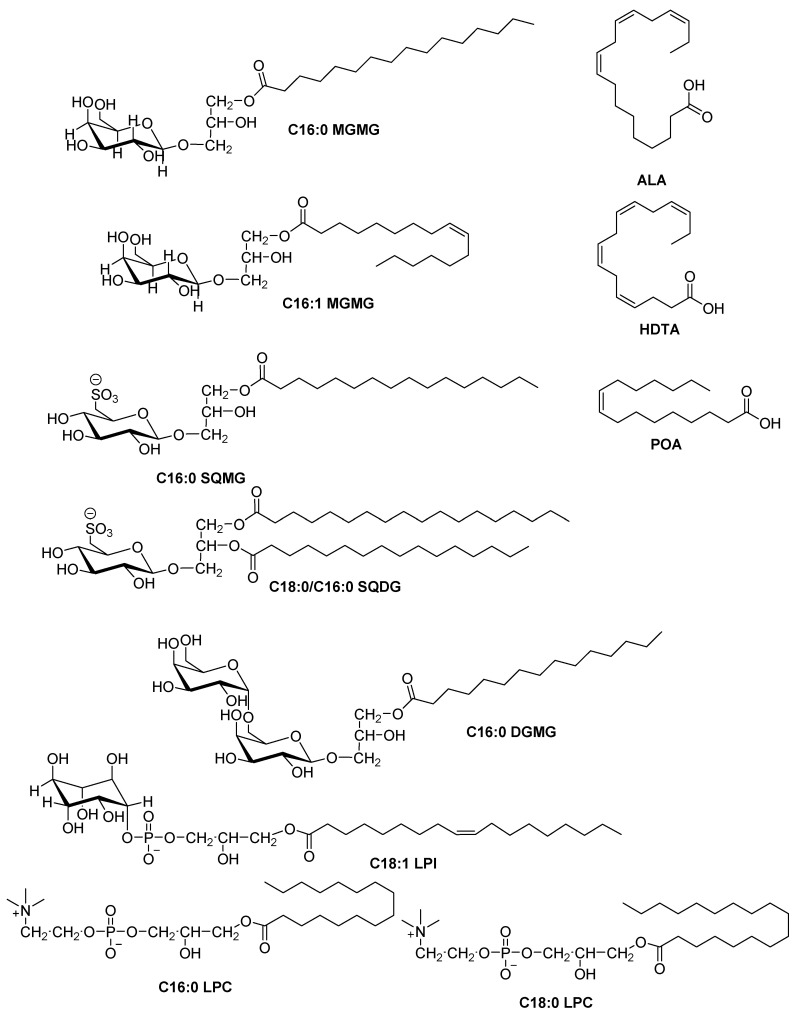
Chemical structure of compounds identified as responsible for antibiotic and antibiofilm activities. In SQDG, the acyl chains can be exchanged.

Therefore, the elucidated compounds with antibacterial and antibiofilm activity were (Figure 4): 

### 2.5. Antibiotic and Antibiofilm Activity of α-Linolenic Acid (ALA) and 1-Palmitoyl-sn-glycero-3-phosphocholine (C16:0 LPC)

As C16:0 LPC has not shown antibiotic activity, only the antibiotic activity of ALA was tested against the three microorganisms used, *E. coli*, *S. aureus,* and *C. parapsilosis*. In these experiments, this polyunsaturated fatty acid was only active against the Gram-positive bacterium (*S. aureus*) and, to a lesser extent, against the yeast (*C. parapsilosis*) (Table 3).

The antibiofilm activities of ALA and C16:0 LPC were tested against all the microorganism strains used in the antibiofilm screening.

A species-dependent effect on biofilm inhibition was observed. Thus, the antibiofilm activity in *K. pneumoniae*, *E. coli*, *S. epidermidis,* and *C. parapsilosis* is mainly due to the presence of C16:0 LPC in a higher proportion or alone. On the contrary, the antibiofilm activity in *C. albicans* is due to the presence of a higher proportion of ALA, and the antibiofilm activity in *S. aureus* and *P. aeruginosa* is due to the presence of ALA alone and when it is also mixed with C16:0 LPC but at lower concentrations (Table 4). The activities are comparable with the minimum biofilm inhibitory concentrations (MBICs) obtained with the corresponding extracts. 

## 3. Discussion

Microalgae are organisms of great interest due to their ability to synthesize biologically active compounds, their rapid growth, allowing a high availability of biomass, and the possibility of adjusting their biochemical composition depending on the cultivation conditions [55]. Thus, the use of microalgae, as well as cyanobacteria, has become increasingly important for humans due to their potential antimicrobial properties, among others. The compounds associated with these bioactivities are normally produced in small quantities from microalgae because most of them are secondary metabolites. However, lipids are one of the primary metabolites of microalgae and cyanobacteria, which enrich their utility in the form of food, pharma, and fuels.

In this work, two strains of the chlorophyta *Scenedesmus brasiliensis*, ACOI 1261 and ACOI 565, were studied for their production of antibiotic lipids and, specifically, in this case, HDTA and ALA fatty acids (Table 1). The ability of the *Scenedesmus* genus to produce antibiotic molecules of a lipid nature, similar to the antibiotic fatty acids identified in this work, has been described previously; specifically, HDTA was also isolated from a lipid extract of *Scenedesmus obliquus* rich in different fatty acids, such as palmitic, oleic, or linolenic acids. The broad-spectrum antibiotic activities of HDTA have been reported, although its major activity is focused on Gram-positive bacteria [56], as is the case in this work (*S. aureus* bactericidal activity). This polyunsaturated fatty acid is not exclusive to microalgae [57] since it has also been identified in marine bacteria, such as *Pseudoalteromonas* sp., showing an attenuating effect on *Vibrio alginolyticus* biofilms [58]. Similarly, the unusual polyunsaturated fatty acid hexadeca-6,9,12-trienoic acid (structurally very similar to HDTA) isolated from the diatom *Phaeodactylum tricornutum* was found to be active against Gram-positive bacteria (*S. aureus*), as well as against the Gram-negative marine pathogen *Listonella anguillarum* [35]. 

Two other microalgae species, *Chlorella vulgaris* and *Scenedesmus obliquus,* are rich producers of ALA, with this polyunsaturated fatty acid representing up to 22% of the total fatty acids content [26]. In addition, potent antimicrobial activities against a wide range of Gram-positive bacteria, such as *Listeria monocytogenes*, *B. cereus,* and *S. aureus,* have been attributed to this fatty acid [59,60,61], as well as against Gram-negative bacteria, such as *Helicobacter pylori* [62]. Its inhibitory effect on fatty acid biosynthesis in *S. aureus* is well-known [24] and may explain the bactericidal activity of this polyunsaturated fatty acid found to be effective against this Gram-positive bacteria in this work (Table 1 and Table 3). ALA also presents moderate antifungal activity against *C. parapsilosis,* although to a lesser extent than in the case of *S. aureus* (Table 3). On the other hand, it has been reported that ALA was able to inhibit enzymes, such as bacterial enoyl–acyl carrier protein reductase (FabI), necessary for fatty acid biosynthesis within the plasma membrane [12,19,20,21]. These mechanisms of action may explain why Gram-positive bacteria are traditionally more sensitive than Gram-negative bacteria (the MIC value of ALA in the case of *E. coli* was higher than 250 mg/L, Table 3), which possess an outer membrane, making the penetration and bactericidal effect of these compounds more difficult [12,26,41]. In this work, the antibiotic fatty acid ALA was also identified and isolated from the cyanobacterium *Sphaeropermopsis* sp. LEGE 00249 (Table 1).

Another unsaturated fatty acid, POA, is known to be the main antimicrobial fatty acid that protects against *S. aureus* causing skin infections in mammals [63]. Fatty acids-based microemulsions based on POA were able to inhibit the growth of *S. aureus* [64], and this fatty acid has shown to have a potent antibacterial effect against *Streptococcus* sp. and *Neisseria gonorrhoeae* [65,66]. POA from the diatom *Phaeodactylum tricornutum* was active against MRSA [37,38]. Recently, it has been shown that POA was able to alter the *Acinetobacter baumannii* quorum sensing (QS) communication system by decreasing the QS regulator AbaR, which decreased biofilm formation by up to 38% [67]. Extracts of the cyanobacterium *Synechocystis* sp. rich in POA were active against *S. aureus* [68]. In this work, POA was also identified and isolated from another cyanobacteria strain, *Sphaerospermopsis* sp. LEGE 00249. POA is a poor substrate for phospholipid biosynthesis and consequently accumulates in the cell, with a deleterious effect on metabolism [69]. The results presented are in line with those of previous studies, showing antibacterial activity against *S. aureus*. 

As far as GLs are concerned, several studies have identified fractions of these compounds from algae with antimicrobial activity, but it was only possible to isolate and characterize the main compounds responsible for this activity in some cases. A crude extract of seaweed *Fucus evanescens* rich in glycolipids showed strong antibacterial activity against *Hemophilus influenza*, *Legionella pneumophila*, *Cutibacterium acnes,* and *Streptococcus pyogenes*, as well as against *Clostridium difficile* and *S. aureus*. The authors chemically synthesized this major glycolipid (MGDG), but the effect was reduced with respect to the glycolipid-rich fraction, suggesting a possible synergistic effect [70]. On the other hand, three sub-fractions from an organic extract of *Sargassum vulgare* rich in MGDG, DGDG, and SQDG showed inhibitory activity against biofilm-forming marine bacteria *Pseudoalteromonas elyakovii*, *Halomonas marina,* and *Shewanella putrefaciens* at MBIC values of 0.01 μg/mL [71]. MGDG-palmitoyl purified from a methanolic extract of the cyanobacterium *Oscillatoria acuminata* was more effectively active against extended-spectrum beta-lactamase (ESBL)-producing bacteria (*E. coli*, *Stenotrophomonas maltophilia,* and *Enterobacter asburiae*) than fourth-generation cephalosporins. In addition, confocal laser scanning microscopy studies showed that the cell membrane was damaged, leading to cell lysis [72]. In this work, the cell extract from another cyanobacterium, *Sphaerospermopsis* sp. LEGE 00249, as well as extracts from the microalgae *S. brasiliensis* ACOI 1261, gave rise to fractions enriched in MGMGs, also showing antibacterial activity against *S. aureus* (Table 1). Moreover, a glycolipid-rich fraction, the main bioactive lipid of which was MGDG (20:5/16:0), isolated from the red alga *Chondria armata*, showed antibiotic activity against *C. albicans*, *C. neoformans,* and *Klebsiella* sp [73]. 

While in this work no antifungal activity associated with fractions enriched in MGDGs (Table 1) was observed, the structurally similar GLs, SQDG, and SQMG, isolated from the macroalgae *Ulva fasciata* and *Taonia atomaria,* showed high inhibition against *E. coli* and *B. subtilis* [74]. In this work, SQDG was isolated from microalgae (*S. brasiliensis* ACOI 1261) and cyanobacteria (*Sphaerospermopsis* sp. LEGE 00249) extracts (Table 1), with both cases showing antibiotic activity against *S. aureus*. 

Although the antimicrobial activities of lipids have been previously reported in several microorganisms, with MICs above 100 µg/mL [13,20], recent studies suggest that lipids exhibit antibiofilm activity against bacteria and fungi at lower concentrations than their corresponding MIC [17,75]. In our case, a lysophosphatidylcholine (C16:0 LPC) but not fatty acids showed antibiofilm activity against *K. pneumoniae*, *E. coli*, *S. epidermidis,* and *C. parapsilosis*. The effect of C16:0 LPC on *Candida* biofilms has also been studied previously [76], attributing this inhibition activity to the induction of the reactive oxygen species system. The authors found 54% of biofilm inhibition at a concentration of 500 mg/L, whereas an inhibition of about 40–70% at concentrations ≤ 64 mg/L was observed in the present work. C16:0 LPC has also been isolated from the methanolic extract of the cyanobacteria *Oscillatoria subuliformis* [77]. This extract showed a biofilm inhibition activity of 56%. This activity was maintained when the extract was used for coating catheters. The antibiofilm activity of C16:0 LPC was also studied against *Acinetobacter baumannii* [67]. In this case, a 38% of biofilm formation inhibition was observed at a concentration of 0.02 mg/mL. This activity was attributed to the decreased expression of the *abaR* gene in the presence of C16:0 LPC, decreasing *N*-acyl-homoserine lactone production and, thereby, interfering with the QS system. In our case, C16:0 LPC alone was able to inhibit biofilm formation by up to 50% at concentrations between 32 and 120 mg/L depending on the species analyzed. 

ALA is the precursor of other important polyunsaturated fatty acids, such as eicosapentaenoic acid (EPA) and docosahexaenoic acid (DHA), all of which are considered as high value products. The ALA produced by *Scenedesmus* strains showed antibiofilm activity. Pure commercial ALA presented biofilm inhibition activity against *P. aeruginosa*, *S. aureus,* and *C. albicans* at concentrations ≤64 mg/L, with this activity being remarkable in the case of *C. albicans* with an MBIC of 1 mg/L. Other studies have evaluated the antibiofilm activity of ALA isolated from animals and plants. ALA from a *Scolopendra* species showed antibiofilm activity against *C. albicans* at a concentration of 20 mg/L [78]. In addition, ALA isolated from the *n*-hexane extract of a semi-green plant showed antibiofilm activity against *Streptococcus mutants* [79]. The antibiofilm effect of ALA in combination with antibiotics has been tested by Chanda et al. (2017) [80]. They observed antibiofilm activity against *P. aeruginosa* combined with tobramycin mainly due to the ability of ALA to increase the bacterial membrane fluidity, disrupting membrane permeability and enhancing the transport of tobramycin into biofilm cells. In our case, ALA had an inhibitory effect on *P. aeruginosa* biofilm formation being enhanced by the presence of C16:0 LPC. As C16:0 LPC and ALA were elucidated in the composition of several active extracts of this study, the synergistic effect of both as antibiofilm agents was studied. We observed that the effect is specific in each studied species. Thus, *K. pneumoniae*, *E. coli*, *S. epidermidis,* and *C. parapsilopsis* were more susceptible to the C16:0 LPC. On the other hand, *C. albicans* is affected only when ALA is present in the culture media. *P. aeruginosa* showed biofilm inhibition in the presence of ALA, alone or in combination with C16:0 LPC. 

Another example highlighting the importance of lipids as antibiotic and antibiofilm compounds is saw palmetto oil (*Serenoa repens*), composed of more than 90% fatty acids, including lauric acid, myristic acid, palmitic acid, and oleic acid. These oils are able to inhibit biofilm formation by *S. aureus*, *E. coli* O157:H7, and *C. albicans* without affecting their fitness. Transcriptomic analyses showed that lauric and myristic acids repressed the expression of several biofilm-related genes (*csgAB*, *fimH,* and *flhD*) in *E. coli* and hypha cell wall gene *HWP1* in *C. albicans*. In addition, the combined treatment of both fatty acids at a concentration of 20 µg/mL with gentamicin showed a synergistic antibacterial activity on *S. aureus* and *E. coli* [81]. 

## 4. Materials and Methods

### 4.1. Microalgae and Cyanobacteria Strains 

Freshwater strains *Scenedesmus brasiliensis* ACOI 1261, ACOI 565, and *Enallax acutiformis* ACOI 118 were obtained from the Coimbra Collection of Algae (http://acoi.ci.uc.pt). Cultures were grown up to 56 L in multiple 10 L flasks (1:1 *v*/*v*) containing 4 L of M7 medium for 15 days with aeration at 25 °C under a light/dark cycle of 16:8 h and photon irradiation of approximately 50 μmol m^−2^ s^−1^ for biomass extraction and subsequent isolation of compounds. Cultures were harvested after reaching the stationary phase by centrifugation (Thermo Scientific Megafuge 8, 4500 rpm, 15 min) and then freeze-dried. The lyophilized biomass (~10 g) was disrupted using a ceramic mortar previously exposed to liquid nitrogen followed by a sequential extraction with hexane, ethyl acetate, and methanol. Extraction was performed by adding 4 × 500 mL of each solvent, followed by vortex and centrifugation at 4500 rpm for 15 min. The supernatant was collected, transferred to glass vials, and dried completely in a rotary evaporator (Table 5). The extracts were stored under cold, dark conditions until analysis to avoid hydrolysis of the bioactive molecules.

The cyanobacterium strain *Sphaerospermopsis* sp. LEGE 00249 was obtained from LEGE CC [27] (accession number: KC989701, 16S rRNA gene). The strain was cultured up to 50 L in Z8 medium [28] at 25 °C with constant aeration with a photoperiod of 14 h/10 h light and dark, respectively, and at light intensity of 10–30 µmols photons m^−2^ s^−1^. At the exponential phase, cells were harvested through centrifugation, then frozen and freeze-dried. The biomass (7.7 g d.w.) was sequentially extracted with hexane, ethyl acetate, and methanol (Table 6). 

### 4.2. Microbial Strains

Antibiotic assays were performed against a Gram-positive bacteria(*Staphylococcus aureus* S54F9, *spa* type t1333) [82], a Gram-negative bacteria (*Escherichia coli* AR, collected from urine at the Hospital Clinic of Barcelona), and a fungus (*Candida parapsilosis* SMI416, non-biofilm-forming clinical isolate from a bloodstream infection) [83].

Biofilm inhibition of these extracts was assayed against Gram-negative strains of *E. coli*; *Klebsiella pneumoniae*, *Enterobacter cloacae,* and *Pseudomonas aeruginosa;* Gram-positivestrains of *S. aureus*, Coagulase-negative *Streptococcus*, and *S. epidermidis;* and *C. albicans* and *C. parapsilosis* fungal strains.

### 4.3. Antibiotic Analysis

The three microbial pathogens (*E. coli*, *S. aureus,* and *C. parapsilosis*) were inoculated in 5 mL Mueller–Hinton broth (Oxoid) from glycerol stocks, incubated overnight at 37 °C with agitation at 250 rpm, and diluted in Mueller–Hinton broth (MHB) up to the desired cell density. The final concentration in the microtiter 96-well U-bottom plates (ThermoScientific) was 5 × 10^5^ colony-forming units (CFU)/mL for *S. aureus* and *E. coli*, and 2.5 × 10^5^ CFU/mL for *C. parapsilosis*. 

When SPE-fractions and HPLC sub-fractions were tested for bioactivity-guided fractionation purposes, no serial dilutions were performed (yes/no method). Briefly, 50 μL of each SPE-fraction or sub-fraction resuspended in 14% MeOH in water (*v*/*v*) were mixed with 50 μL of the microorganism suspension in a microtiter plate and incubated overnight at 37 °C statically. Growth controls (broth with bacterial inoculum, without bioactive molecules) as well as sterility (broth only) and solvent controls (bacterial inoculum with a final concentration of 7% MeOH in water *v*/*v*) were also included.

When the antibiotic activity of ALA was studied, an MIC assay was carried out following the guidelines of the Clinical and Laboratory Standards Institute (CLSI) [84]. A stock solution of ALA in 0.15 M aqueous solution of tris-HCl pH 8.5 was prepared and serial two-fold dilutions ranging from 250 mg/mL to 0.244 mg/mL were carried out in a microtiter plate with 50 µL of sterile Milli-Q water pre-added to all the wells. 50 µL of the corresponding microorganism suspension in 2x MHB was added to all the wells. Growth, sterility, and solvent controls were also included. 

In all the cases, microbial sedimentation was checked by visual verification, and each experiment was performed in duplicate. The minimum bactericidal/fungicidal concentration (MBC/MFC) was determined according to the CLSI protocol by plating 20 μL from each well, showing no visible growth at 24 h, onto a solid medium. For that purpose, the microtiter plate was replicated onto a selective/differential solid medium, such as mannitol salt agar (MSA, VWR Chemicals) for *S. aureus*, eosin methylene blue (EMB, VWR Chemicals) for *E. coli*, and Sabouraud agar (VWR Chemicals) for *C. parapsilosis,* with a 96-pin replicator in order to distinguish between bacteriostatic and bactericidal activities. 

### 4.4. Antibiofilm Analysis

The antibiofilm assay was performed by the broth microdilution assay described in the CLSI document M7-A7 [84] with some modifications described by Cepas et al. [8]. 

The results are expressed as percentage of biofilm inhibition observed in comparison with the biofilm of the bacterial strain without extract. No MBIC values could be obtained because the concentration of each compound in each fraction was not known. 

The MBICs of both ALA and 1-Palmitoyl-*sn*-glycero-3-phosphocholine (C16:0 LPC) were tested by serial dilutions from a concentration of 128 mg/L to 0.25 mg/L against nine strains representing Gram-negative, Gram-positive, and fungi species (*K. pneumoniae, E. coli, P. aeruginosa, Enterobacter cloacae, S. aureus*, Coagulase Negative Streptococcus, *S. epidermidis, C. parapsilosis,* and *C. albicans*). Synergies between the two compounds were also studied by the determination of the MBIC using mixtures of different proportions of both compounds (1:0, 1:1, 1:0.5, 0.5:1, 0:1).

### 4.5. Solid Phase Extraction Methods for Methanolic and Ethyl Acetate Extracts

All the methanolic extracts (fraction code C) from microalgae strains (ACOI 565, ACOI 118) were submitted to several non-retentive solid phase extractions (Phenomenex Strata^®^ C18-E, 55 μm, 10 g, 60 mL, C18 Cartridges) and developed ad hoc to reduce the complexity of the samples as well as to eliminate column killers that could compromise further purification steps. Hence, the analytes of interest were eluted, and the interferences were retained in the sorbent. A maximum of 10 mL of the microalgae extracts sample reconstituted in MeOH 20% (*v*/*v*) was added to the conditioned sorbent and, for the elution (4 to 8 bed volumes each), a gradient solvent of H_2_O (A) and MeOH (B) from 20 to 100% B in 10% steps (C1–C9) followed by a washing step with acetone (C10) was used. All the SPE fractions were dried out under vacuum in a rotavapor (RV 10 Digital, IKA) equipped with a vertical condenser maintained at −10 °C (RC-10 Digital Chiller, VWR).

The ethyl acetate extract (fraction code B) from the ACOI 1261 strain was submitted to SPE (Sep-Pak^®^ Waters^®^ Silica, 55 μm, 5 g, 20 cc, vac cartridge). In this case, a maximum of 5 mL of the microalgae extract reconstituted in EtOAc/Hex 20:80 (*v*/*v*) was added to the conditioned stationary phase and 4-8 bed volumes were used for the stepwise elution, which was performed as follows: from EtOAc/Hex 20:80 (*v*/*v*) to EtOAc 100% in 10% steps (B1-B9) followed by a washing step with MeOH/EtOAc 25:75 (*v*/*v*) (B10). Each SPE fraction was dried out as described previously.

### 4.6. Bioassay-Guided HPLC-DAD Purification

The active SPE fractions in antibiotic and/or antibiofilm tests were selected for further purification and filtered through 0.8 and 0.2 μm filters (Acrodisc^®^, Pall, NY, USA). For each fraction, a customized analytical HPLC program that assured the best peak resolution in 30 min was designed, and an Agilent 1260 Inifinity LC system equipped with an analytical reversed-phase RP-18 HPLC column (250 × 4.0 mm, Pursuit^®^ XRs, 5 μm, Agilent Technologies) was used for that purpose. The column was coupled to a UV detector set to 255 and 280 nm at a flow rate of 1 mL/min, and 50 μg of sample was injected. The corresponding analytical program was scaled up to semi-preparative conditions with the Waters Gradient Calculator (Waters Corporation, Milford, MA, USA). At this purification step, a semipreparative RP-18 HPLC column (250 × 10.0 mm, Pursuit XRs, 5 μm, (Agilent Technologies, Santa Clara, CA, USA) with a guard column (50 × 10.0 mm, Pursuit^®^ XRs, 5 μm, Agilent Technologies) was used. The flow rate was increased up to 4 mL/min over a period of 47 minutes and sub-fractions were collected every 30 seconds (94 sub-fractions in total, 2 mL per fraction) in a 96-well DeepWell plates (ThermoScientific, Waltham, MA, USA). Wells 95 and 96 were empty to remain solvent and negative controls, respectively, in antibiotic and antibiofilm assays. As a general rule, a maximum of 50 mg was used in each semipreparative injection in order to avoid detector saturation; therefore, on many occasions, several iterative rounds of purification were needed. Under both analytical and semipreparative conditions, H2O (A) and MeCN (B) were used as mobile phases, both with 0.1% (*v*/*v*) of formic acid. 

The semipreparative programs for the different SPE fractions with antibiotic and/or antibiofilm activity were as follows. For the ACOI565_C4 extract (27 mg): 0–3.13 min (25% B), 3.13-37.5 min (25–100% B), 37.5–40.63 min (100% B), 40.63–42.19 min (100-25% B), and 42.19–47 min (25% B); for the ACOI565_C8 extract (129.1 mg): 0–3.13 min (40% B), 3.13–31.25 min (40–100% B), 31.25–37.5 min (100% B), 37.5–39.06 min (100–40% B), and 39.06–47 min (40% B); for the ACOI1261_C8 extract (116.5 mg): 0–1 min (35% B), 1–3 min (35–60% B), 3–9 min (60–85% B), 9–32 min (85–100% B), 32–40 min (100% B), 40–41 min (100–35%), and 41–47 min (35% B); and for the ACOI118_C6 extract (9 mg): 0–3 min (60% B), 3–31 min (60–100% B), 31–40 min (100% B), 40–41 min (100–60% B), and 41–47 min (60% B). 

In the case of the methanol extract of the cyanobacterium *Sphaerospermopsis* sp. LEGE00249, an SPE was also performed, followed by a semi-preparative HPLC-DAD fractionation, as described in [9]. Briefly, a first round of fractionation gave rise to three groups of bioactivities, and the first group (group A, 14 mg) was pooled and subjected to a second round of purification, obtaining 94 new sub-fractions. The complete list of isolated fractions and sub-fractions amounts is described in Table 7.

### 4.7. HPLC-HRESIMS Analyses

The HPLC active sub-fractions and inactive flanking sub-fractions were injected into HPLC-HRESIMS equipment to decipher which *m*/*z* ions were responsible for the detected bioactivity. For that purpose, a UPLC system (Dionex Ultimate 3000, ThermoScientific) coupled to an ESI-UHR-Qq-TOF Impact II spectrometer (Bruker), which acquired data in the negative or positive ion mode, with a *m*/*z* range from 40 to 2000 Da, was used. For the chromatographic separation, elution was performed through an analytical RP-18 HPLC column (50 × 2.1 mm, Zorbax® Eclipse Plus, 1.8 μm, Agilent Technologies) with a combination of H_2_0 (A) and MeCN (B), both with 0.1% (*v*/*v*) of formic acid. Analytes were eluted at a flow rate of 0.25 mL/min under the following conditions: 0–1 min (10% B), 1–4 min (10–35% B), 4–5 min (35% B), 5–8 min (35–100% B), 8–10 min (100% B), 1-11 min (100–10% B), and 11–15 min (10% B). Data were analyzed using Compass DataAnalysis 4.3 (Bruker).

### 4.8. NMR Assays

All the ^1^H, ^13^C{^1^H}, ^31^P{^1^H}, and 2D NMR spectra were recorded on a Bruker Avance III HD 600 MHz NMR (14.0 T) spectrometer equipped with a QCI-P CryoProbe™ (proton-optimized quadruple resonance NMR ‘inverse’ probe). The samples were prepared by dissolving the microalgae and cyanobacteria extracts in 0.5 mL of methanol-*d_4_*. Key parameters for the acquisition of 1D and 2D NMR spectra were the same as previously reported [57,85]. TopSpin 3.6 (Bruker) was used for acquiring and processing the NMR spectra.

### 4.9. Reagents and Biochemicals

All solvents used for SPE extraction, HPLC-DAD purification, and mass spectrometry analysis were LC-MS grade from either Sigma-Aldrich or VWR Chemicals. Authentic ALA was purchased from Cayman Chemical, and 1-palmitoyl-*sn*-glycero-3-phosphocholine (C16:0 LPC) was purchased from Sigma-Aldrich.

## 5. Conclusions

Cyanobacteria and microalgae produce a great variety of lipids with antibiotic and antibiofilm activity against the most important pathogens causing severe infections in humans. Therefore, their use in clinical treatments alone or in combination with well-known antibiotics requires further investigation as an alternative to the current treatments. However, further studies are needed to determine the in vivo efficacy of these lipids, their mechanisms of action, and their availability in the amounts required. 

## Figures and Tables

**Table 1:** Antibiotic activity.

Microalgae/Cyanobacteria Strain	SPE-Fraction	HPLC Sub-Fraction	Molecules Detected	Microorganism
**ACOI 1261**	B8	F52	C16:0 MGMG, C18:0/C16:0 SQDG	*S. aureus*
**ACOI 118**	C6	F39	C16:0 SQMG	*S. aureus*
**ACOI 565**	C8	F67	ALA	*S. aureus*
	C4	F68	HDTA	*S. aureus*
**LEGE 00249**	-	F34 (from Group A F69-F76 refractionation)	C16:1 MGMG	*S. aureus*
	-	F48 (from Group A F69-F76 refractionation)	C16:0 MGMG	*S. aureus*
	-	F83	ALA, C16:0/C18:0 SQDG	*S. aureus*
	-	F86	POA	*S. aureus*

SPE, solid phase extraction; HPLC-DAD, high-performance liquid chromatography with diode-array detection; SQDG, sulfoquinovosyl–diacylglycerol; MGMG, monogalactosyl–monoacylglycerol; SQMG, sulfoquinovosyl–monoacylglycerol; ALA, α-linolenic acid; HDTA, hexadeca-4,7,10,13-tetraenoic acid; POA, palmitoleic acid; B, ethyl acetate fraction; C, methanol fraction; F, sub-fraction.

**Table 2:** Antibiofilm activity.

Microalgae/Cyanobacteria Strain	SPE-Fraction	HPLC Sub-Fraction	Molecules Detected	Microorganism	% of Biofilm Inhibition
**ACOI 1261**	B8	F26-27	C16:0 MGMG, C18:0/C16:0 SQDG	CoNS	80%
		F28-29	C16:0 MGMG, C18:0/C16:0 SQDG	CoNS *C. parapsilosis*	40% 40%
		F47-51	C16:0 MGMG, C18:0/C16:0 SQDG	*E. coli* CoNS	80% 40%
		F59-66	C16:0 MGMG, C18:0/C16:0 SQDG	*E. coli* *C. parapsilosis*	40% 40%
**ACOI 118**	C6	F36-38	C16:0 SQMG	CoNS	34%
**ACOI 565**	C8	F48-49	C16:0 DGMG C18:0 LPC, C16:0 LPC, C18:1 LPI C16:0 SQMG	CoNS *E. coli* *C. albicans*	60% 60% 60%
**LEGE 00249**	-	F34 (from Group A F69-F76 refractionation)	C16:1 MGMG	CoNS	61%
		F48 (from Group A F69-F76 refractionation)	C16:0 MGMG	CoNS	70%

SPE, Solid Phase Extraction; HPLC-DAD, high-performance liquid chromatography with diode-array detection; SQDG, sulfoquinovosyldiacylglycerol; MGMG, monogalactosylmonoacylglycerol; DGMD, digalactosylmonoacylglycerol; SQMG, sulfoquinovosyl–monoacylglycerol; LPC, lysophosphatidylcholine; B, ethyl acetate fraction; C, methanol fraction; F, sub-fraction.

**Table 3:** Antibiotic activity of ALA.

Microbial Strain	MIC (mg/L)
***E. coli***	>250
***S. aureus***	15.6
***C. parapsilosis***	125

MIC, minimum inhibitory concentration.

**Table 4:** Synergies of antibiofilm activity between C16:0 LPC and ALA.

	50% Biofilm Inhibition				
Microbial Strain	ALA (mg/L)	ALA 1: C16:0 LPC 1 (mg/L)	ALA 1: C16:0 LPC 0.5 (mg/L)	ALA 0.5: C16:0 LPC 1 (mg/L)	C16:0 LPC (mg/L)
***K. pneumoniae***	>128	>128	>128	2	32
***P. aeruginosa***	32	64	32	16	>128
***E. coli***	>128	>128	128	64	32
***E. cloacae***	128	128	128	128	128
**CoNS**	128	128	128	128	128
***S. epidermidis***	128	128	128	64	64
***S. aureus***	64	128	128	64	>128
***C. parapsilosis***	128	128	64	64	64
***C. albicans***	1	8	64	>128	128

ALA, α-linolenic acid; C16:0 LPC, 1-palmitoyl-*sn*-glycero-3-phosphocholine.

**Table 5:** Extract yields of the ACOI strains.

Microalgae Strain	Hexane (mg)	Ethyl Acetate (mg)	Methanol (mg)
**ACOI 1261**	136	69	1109
**ACOI 118**	301	214	821
**ACOI 565**	292	143	1316

**Table 6:** Extract yields of LEGE CC strain.

Cyanobacterium Strain	Hexane (mg)	Ethyl Acetate (mg)	Methanol (mg)
**LEGE 00249**	66.07	352.88	949.65

**Table 7:** SPE fractions and HPLC-DAD sub-fractions yields.

Microalgae/Cyanobacteria Strain	SPE-Fraction	HPLC Sub-Fraction
**ACOI 1261**	B8 (4.3 mg)	F52 (0.7 mg)
		F26–27 (0.5 mg)
		F28–29 (0.7 mg)
		F47–51 (0.9 mg)
		F59–66 (1.1 mg)
**ACOI 118**	C6 (9 mg)	F39 (0.3 mg)
		F36–38 (0.4 mg)
**ACOI 565**	C8 (129.1 mg)	F67 (5.4 mg)
		F48–49 (2.4 mg)
	C4 (27.9 mg)	F68 (0.5 mg)
**LEGE 00249**	-	F34 (0.4 mg)
		F48 (0.4 mg)
